# Effects of exogenous glycine betaine and cycloleucine on photosynthetic capacity, amino acid composition, and hormone metabolism in *Solanum melongena* L.

**DOI:** 10.1038/s41598-023-34509-w

**Published:** 2023-05-10

**Authors:** Tianhang Niu, Jing Zhang, Jing Li, Xiaoping Gao, Hongyan Ma, Yanqiang Gao, Youlin Chang, Jianming Xie

**Affiliations:** 1 College of Horticulture, Gansu Agricultural University, Yingmen Village, Anning District, Lanzhou, 730070 China; 2 Lanzhou New Area Agricultural Science and Technology Development Co., Ltd., Lanzhou, 730000 China

**Keywords:** Plant sciences, Plant physiology

## Abstract

Although exogenous glycine betaine (GB) and cycloleucine (Cyc) have been reported to affect animal cell metabolism, their effects on plant growth and development have not been studied extensively. Different concentrations of exogenous glycine betaine (20, 40, and 60 mmol L^−1^) and cycloleucine (10, 20, and 40 mmol L^−1^), with 0 mmol L^−1^ as control, were used to investigate the effects of foliar spraying of betaine and cycloleucine on growth, photosynthesis, chlorophyll fluorescence, Calvin cycle pathway, abaxial leaf burr morphology, endogenous hormones, and amino acid content in eggplant. We found that 40 mmol L^−1^ glycine betaine had the best effect on plant growth and development; it increased the fresh and dry weight of plants, increased the density of abaxial leaf hairs, increased the net photosynthetic rate and Calvin cycle key enzyme activity of leaves, had an elevating effect on chlorophyll fluorescence parameters, increased endogenous indoleacetic acid (IAA) content and decreased abscisic acid (ABA) content, and increased glutamate, serine, aspartate, and phenylalanine contents. However, cycloleucine significantly inhibited plant growth; plant apical dominance disappeared, plant height and dry and fresh weights decreased significantly, the development of abaxial leaf hairs was hindered, the net photosynthetic rate and Calvin cycle key enzyme activities were inhibited, the endogenous hormones IAA and ABA content decreased, and the conversion and utilization of glutamate, arginine, threonine, and glycine were affected. Combined with the experimental results and plant growth phenotypes, 20 mmol L^−1^ cycloleucine significantly inhibited plant growth. In conclusion, 40 mmol L^−1^ glycine betaine and 20 mmol L^−1^ cycloleucine had different regulatory effects on plant growth and development.

## Introduction

Plant growth and development can be influenced by many factors, such as genetic factors^1^, growing environment^2,3^ and cultivation practices^4^. The primary (sugars, amino acids, nucleotides, etc.) and secondary metabolites (hormones, pigments, and toxins) synthesized by plants can also regulate their growth and development^5,6^. Indoleacetic acid (IAA), abscisic acid (ABA), and gibberellins (GA) can directly participate in and regulate plant growth and development, affecting plant morphological composition and various types of organ differentiation^7,8^. However, alkaloids and non-protein amino acids affect plant growth and development by influencing metabolic pathways^9^. As an important RNA modification, m6A methylation is widely involved in mRNA degradation^10^, splicing, export, stability^10^, and translation^11^, which in turn affects the synthesis and utilization of secondary metabolites in plant^12^. Studies on animal cells have shown that glycine betaine (GB) can affect intracellular m^6^A methylation modifications. However, this has not been reported for plants^13–15^.

Glycine betaine (GB) (N, N, N-trimethylglycine) is a secondary metabolite widely found in plants and animals and is an important osmoregulatory substance^2^. In plant cells, serine is synthesized into GB in the chloroplasts via betaine aldehyde, choline, and ethanolamine^16^. It is a safe and non-toxic class IV organic compound that stabilizes the structure and efficiency of PS II in chloroplasts, which in turn stabilizes or improves photosynthetic efficiency^17^. Similarly, when GB accumulates in plants, the nitrogen therein promotes seed germination and root growth, promoting plant growth^18^. Current research reports on betaine in plants have mainly focused on alleviating the effects of abiotic stresses in plants, such as salt^19^, drought^20^, and heavy metal^21^. This effect has also been verified in studies on mung beans^22^, tomatoes^23^, and zucchini^24^.

Studies have shown that exogenous spraying of GB can stabilize photosynthetic pigments, the net photosynthetic rate, and chlorophyll fluorescence, promote plant growth, increase leaf number, and improve chlorophyll content, etc.^25,26^. Betaine helps to maintain the structural and functional integrity of 1,5-bisphosphate ribulose carboxylase (RuBisCO), RuBisCO-activating enzyme (RCA), fructose-1,6-bisphosphatase (FBPase), FBP aldolase, and PRKase^27^. After betaine accumulates in plants under normal conditions, it can also increase the number of plant seeds and fruits, improve yield, and increase the number of flowers^28^. However, published results showed that eggplant did not accumulate betaine on its own when subjected to abiotic stresses; therefore, exogenous application of betaine was necessary^29^.

Cycloleucine (Cyc), a chemical inhibitor, has mostly been studied in animals and microorganisms. In animal cell assays, its main action was on methionine adenosyl transferase, which can reduce the level of S-adenosylmethionine in cells. It has been shown that high concentrations of cycloleucine are cytotoxic and can affect cell development^30^. In *Escherichia coli* cultures, adding cycloleucine to the medium can affect cell metabolism^31^. In recent years, most applications of cycloleucine have focused on m^6^A methylation in animals, and it has been widely used as a methylation inhibitor. However, its effects on plant growth and development have rarely been reported. In plants, the exogenous administration of cycloleucine strongly inhibits light-induced activation of catalase mRNA^31^. Plant growth was slowed, and chlorosis and even necrosis were observed after the exogenous application of cycloleucine during the cultivation of potato detoxification seedlings^32^, further confirming that cycloleucine is toxic not only to animal cells but also to plant cells.

Eggplant (*Solanum melongena* L.) is the fifth largest cash crop in solanaceous plants cultivated worldwide after potato, tomato, pepper, and tobacco^33^. Eggplants are rich in various nutrients and trace elements, such as plant polyphenols, which help protect cell membranes and enhance memory function in the brain^34^. The antioxidant content of eggplant also reduces the risk of cancer^35^ and prevents cardiovascular disease^36^. The antioxidant content of eggplant also reduces the risk of cancer and prevents cardiovascular diseases^36^. Most recent studies on the effects of exogenous GB on eggplant have focused more on abiotic stress amelioration and less on the growth and development of seedlings.

This study aimed to investigate the effects of exogenous spraying of different concentrations of glycine betaine and cycloleucine on the growth and development of eggplant seedlings and to enhance our understanding of the underlying mechanisms by which glycine betaine and cycloleucine influence plant growth and development. And to screen reasonable exogenous glycine betaine and cycloleucine concentrations for subsequent m6A methylation assays.

## Results

### Effect of exogenous glycinebetaine and cycloleucine on growth parameters in eggplant

**Figure 1:**
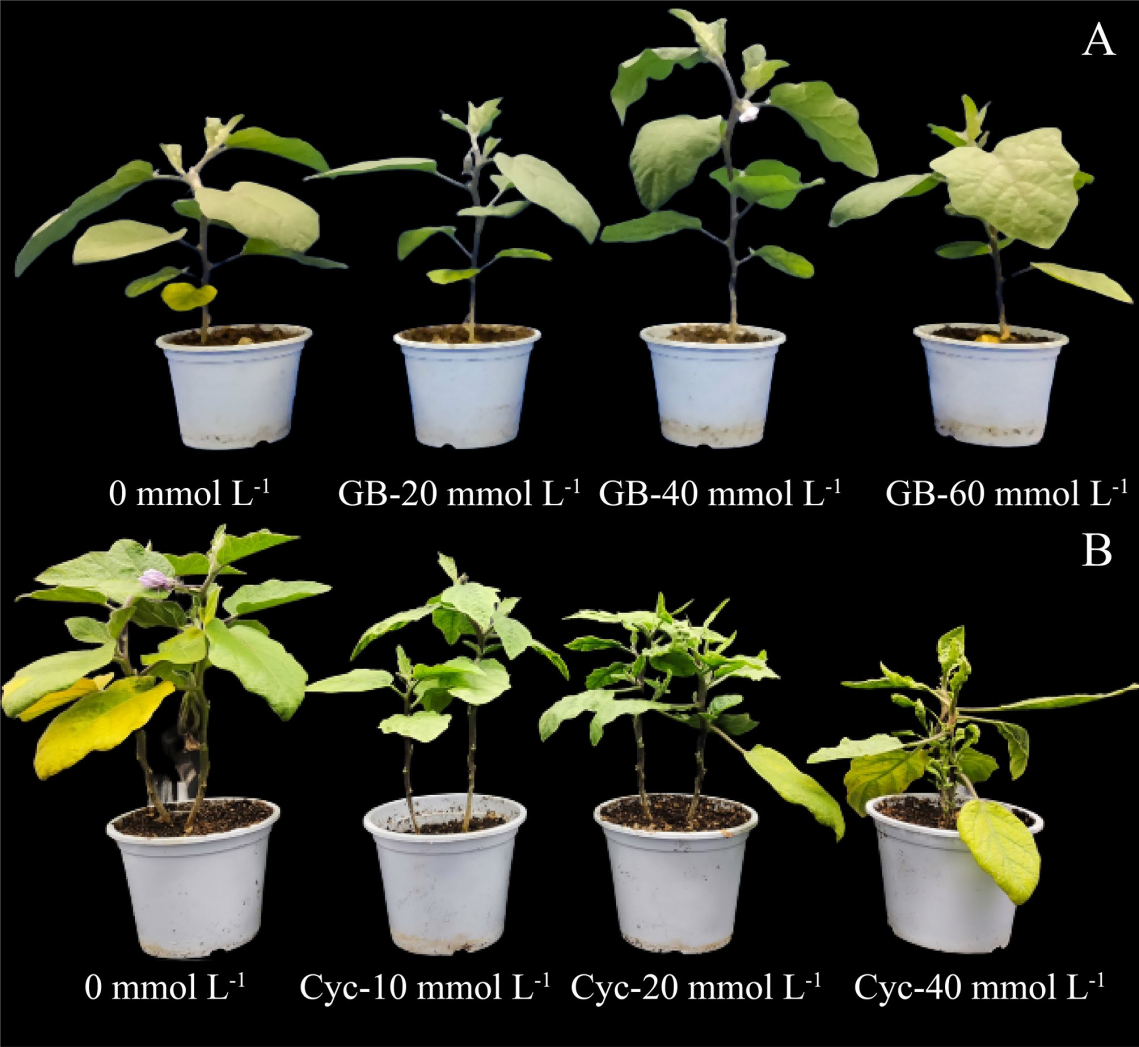
Effect of exogenous glycine betaine and cycloleucine on growth attributes of eggplant. (**a**) the effect of glycine betaine on growth attributes of eggplant. (**b**) the effect of cycloleucine on growth attributes of eggplant.

Exogenous GB promoted eggplant growth and flowering (Fig. 1A). Compared with 0 mmol L^−1^ (Table 1), exogenous GB at 40 mmol L^−1^ increased eggplant plant height, stem diameter, shoot fresh weight, shoot dry weight, plant fresh weight, and dry weight by 22.59%, 7.96%, 22.91%, 23.64%, 14.73%, and 19.34%, respectively (*p* < 0.05). However, after treatment with 20 mmol L^−1^ and 60 mmol L^−1^, the height, stem diameter, and shoot dry weight increased, but not significantly compared with the control (0 mmol L^−1^). Therefore, GB may promote the growth of eggplant seedlings at an optimal concentration of 40 mmol L^−1^.

**Table 1:** Effect of exogenous glycine betaine and cycloleucine on growth parameters of eggplant.

	Treatments (mmol L^−1^)	Plant height (cm)	Stem diameter (mm)	Shoot fresh weight (g)	Shoot dry weight (g)	Root fresh weight (g)	Root dry weight (g)	Fresh Weight of plant (g)	Dry Weight of plant (g)
GB	0	16.69 ± 0.45b	5.15 ± 0.08b	10.17 ± 0.29c	2.03 ± 0.07b	7.15 ± 0.21a	0.71 ± 0.04ab	17.11 ± 0.49b	2.74 ± 0.11b
	20	17.40 ± 0.62b	5.30 ± 0.07ab	10.79 ± 0.54bc	2.08 ± 0.12ab	6.14 ± 0.27b	0.61 ± 0.04bc	17.14 ± 0.90b	2.69 ± 0.15b
	40	20.46 ± 0.76a	5.56 ± 0.08a	12.50 ± 0.43a	2.51 ± 0.26a	7.31 ± 0.42a	0.77 ± 0.06a	19.63 ± 0.74a	3.27 ± 0.30a
	60	16.56 ± 1.12b	5.05 ± 0.170b	11.94 ± 0.37ab	2.00 ± 0.07b	5.36 ± 0.23b	0.52 ± 0.04b	17.30 ± 0.51b	2.52 ± 0.09b
Cyc	0	23.03 ± 0.94a	5.84 ± 0.24a	23.11 ± 1.69a	2.69 ± 0.45a	11.87 ± 0.92a	1.17 ± 0.10a	34.97 ± 1.70a	3.86 ± 0.52a
	10	17.93 ± 0.57c	4.92 ± 0.10b	15.48 ± 1.76b	2.00 ± 0.22ab	5.06 ± 0.36d	0.63 ± 0.02b	20.54 ± 1.79b	2.63 ± 0.21b
	20	13.93 ± 0.13b	5.06 ± 0.18b	15.09 ± 2.24b	2.38 ± 0.33ab	7.12 ± 0.58c	1.00 ± 0.13a	22.19 ± 2.34b	3.38 ± 0.42ab
	40	10.77 ± 0.22d	4.93 ± 0.15b	12.07 ± 0.48b	1.74 ± 0.08b	9.10 ± 0.51b	1.22 ± 0.09a	21.17 ± 0.78b	2.97 ± 0.14ab

The growth parameters of eggplant plants were obtained on the 14th day after the end of the treatment. The results are shown as the mean ± SE of three replicates, and the different letters denote significant differences between treatments (*p* < 0.05), according to Duncan's multiple range tests. 0 mmol L^−1^: control.

Exogenous cycloleucine treatment directly resulted in stunted growth with increasing cycloleucine concentrations (Fig. 1B), deformed plant leaves, delayed flower bud differentiation, loss of apical dominance, and an increased number of lateral shoots (Figs. S1–S3). Compared with the control, the plant height, stem diameter, and fresh weights of shoots and roots were significantly decreased in the 10 mmol L^−1^, 20 mmol L^−1^, and 40 mmol L^−1^ treatments (*p* < 0.05).

### Effect of exogenous glycine betaine and cycloleucine on the morphology of leaf abaxial hairs

**Figure 2:**
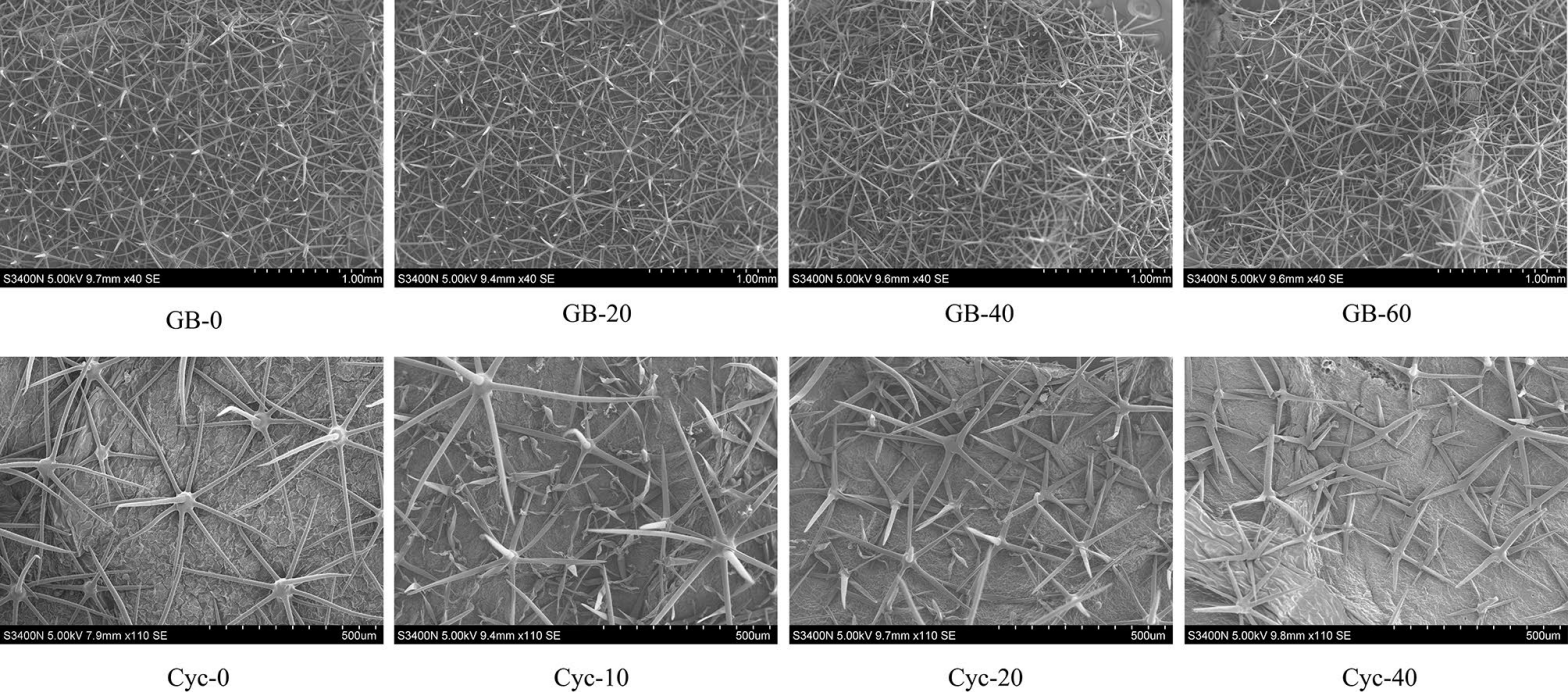
Effect of exogenous glycine betaine and cycloleucine on abaxial hairs of eggplant leaves. GB-0: 0 mmol L^−1^; GB-20: 20 mmol L^−1^ glycine betaine; GB-40: 40 mmol L^−1^ glycine betaine; GB-60: 60 mmol L^−1^ glycine betaine. Cyc-0:0 mmol L^−1^; Cyc-10: 10 mmol L^−1^ cycloleucine; Cyc-20: 20 mmol L^−1^ cycloleucine; Cyc-40: 40 mmol L^−1^ cycloleucine.

Scanning electron microscopy was used to observe the morphology of the abaxial hair of eggplant leaves under different treatments. To observe the change in density of leaf abaxial hairs after exogenous glycine betaine treatment, we chose to magnify the leaf abaxial hairs by 40 times. In order to observe the morphological changes of leaf abaxial hairs after cycloleucine treatment, we chose to magnify the leaf abaxial hairs 110 times. After spraying with GB, the distribution density of leaf abaxial hairs increased with increasing GB concentration compared with the control (Fig. 2). Conversely, the morphology of abaxial leaf hairs was obvious changed in the cycloleucine treatment (Fig. 2 Cyc-0, Cyc-10, Cyc-20, and Cyc-40). The number of abaxial leaf hairs with needle-like projections was significantly less than that of the control, and some of the needle-like projections were curled and almost flattened.

### Effect of exogenous glycine betaine and cycloleucine on gas exchange parameters and chlorophyll content in eggplant

**Figure 3:**
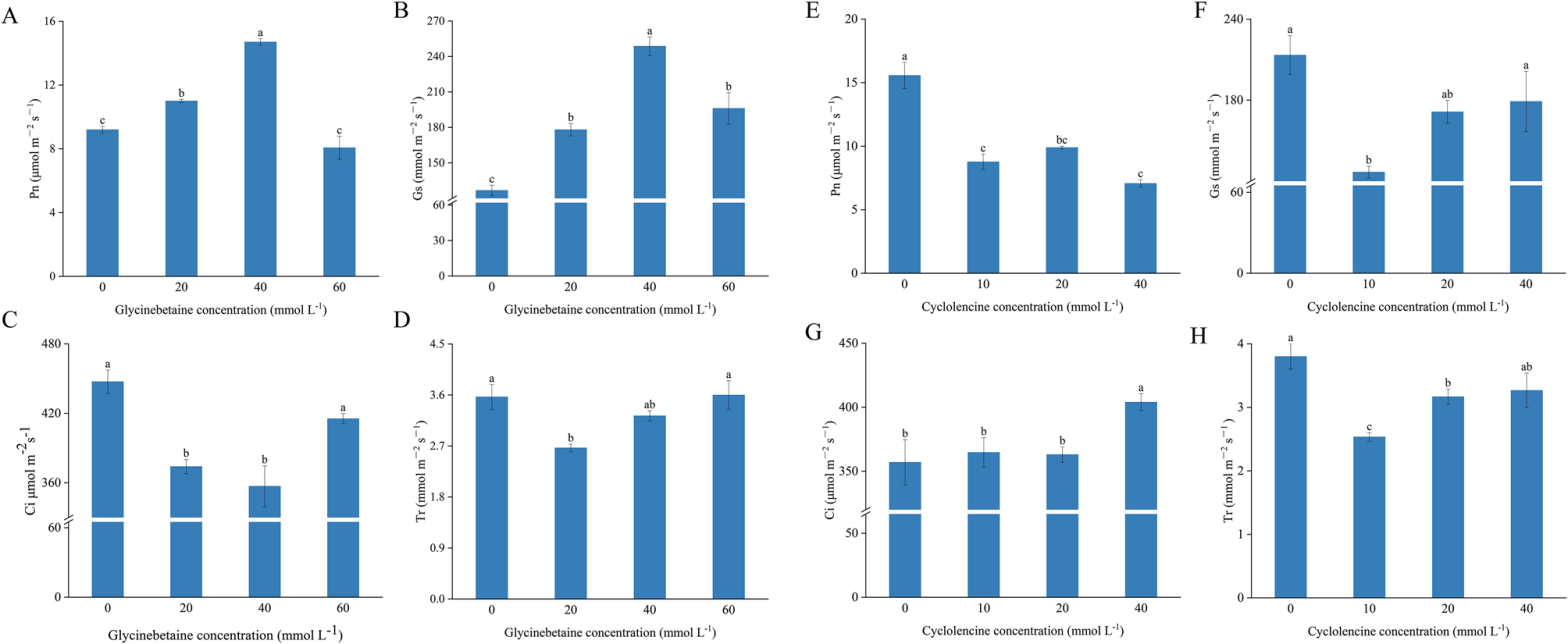
Effect of exogenous glycine betaine and cycloleucine on gas exchange parameters of eggplant leaves. The different letters denote the significant difference among treatments (*p* < *0.05*), according to Duncan’s multiple tests. 0 mmol L^−1^: control.

The gas exchange parameters of eggplant plants treated with exogenous betaine are shown in Fig. 3. The Pn and Gs of plants treated with 20 mmol L^−1^, 40 mmol L^−1^, and 60 mmol L^−1^ GB were significantly higher than those of the 0 mmol L^−1^ (Fig. 3). The increase in these parameters also indicated that spraying exogenous betaine could promote plant growth (Fig. 1A, Table 1), but the Tr (transpiration rate) values of plants treated with 20 mmol L^−1^ GB decreased (*p *< 0.05). Compared to the 20 mmol L^−1^ and 40 mmol L^−1^ treatments, the Pn value of plants treated with 60 mmol L^−1^ GB decreased (*p *< 0.05), indicating that a high GB concentration was not favorable for the Pn value. Similarly, compared with plants treated with 0 mmol L^−1^ cycloleucine, the growth of plants treated with 10 mmol L^−1^, 20 mmol L^−1^, and 40 mmol L^−1^ cycloleucine was significantly inhibited (Fig. 1B), and Pn, Gs, and Tr values decreased (Fig. 3E,F,H). However, the Ci values of the plants treated with 40 mmol L^−1^ cycloleucine were higher than those of the other treatments.

The 40 mmol L^−1^ GB treatment significantly increased the content of chlorophyll a (Chl. a), chlorophyll b (Chl. b), and total chlorophyll (Chl. T) in the leaves by 33.15%, 53.23%, and 37.80%, respectively, compared with the control treatment (Table 2). However, there was no significant difference in the content of Chl. a, Chl. b, and Chl. T between the 60 mmol L^−1^ GB-treated plants and those with the control treatment. Exogenous cycloleucine treatment (all concentrations) significantly decreased the content of Chl. a, Chl. b, and Chl. T compared to the control (Table 2).

**Table 2:** Effect of exogenous glycine betaine and cycloleucine on photosynthetic pigment content of eggplant leaves.

	Treatments (mmol L^−1^)	Chlorophyll a (mg g FW)	Chlorophyll b (mg g FW)	Chlorophyll T (mg g FW)
GB	0	1.84 ± 0.01c	0.62 ± 0.01b	2.46 ± 0.01c
	20	2.00 ± 0.04b	0.77 ± 0.07b	2.77 ± 0.04b
	40	2.45 ± 0.07a	0.95 ± 0.03a	3.39 ± 0.10a
	60	1.91 ± 0.05bc	0.68 ± 0.05b	2.59 ± 0.10bc
Cyc	0	2.22 ± 0.11a	0.75 ± 0.06a	2.23 ± 0.15a
	10	1.51 ± 0.11b	0.50 ± 0.04b	1.57 ± 0.08b
	20	1.01 ± 0.08c	0.35 ± 0.03c	0.82 ± 0.12c
	40	0.80 ± 0.04c	0.26 ± 0.01c	0.55 ± 0.03c

The growth parameters of eggplant plants were obtained on the 14th day after the end of the treatment. The results are shown as the mean ± SE of three replicates, and the different letters denote significant differences between treatments (*p* < 0.05), according to Duncan's multiple range tests. 0 mmol L^−1^: control.

### Effect of exogenous glycine betaine and cycloleucine on Calvin cycle enzyme activity in eggplant leaves

**Figure 4:**
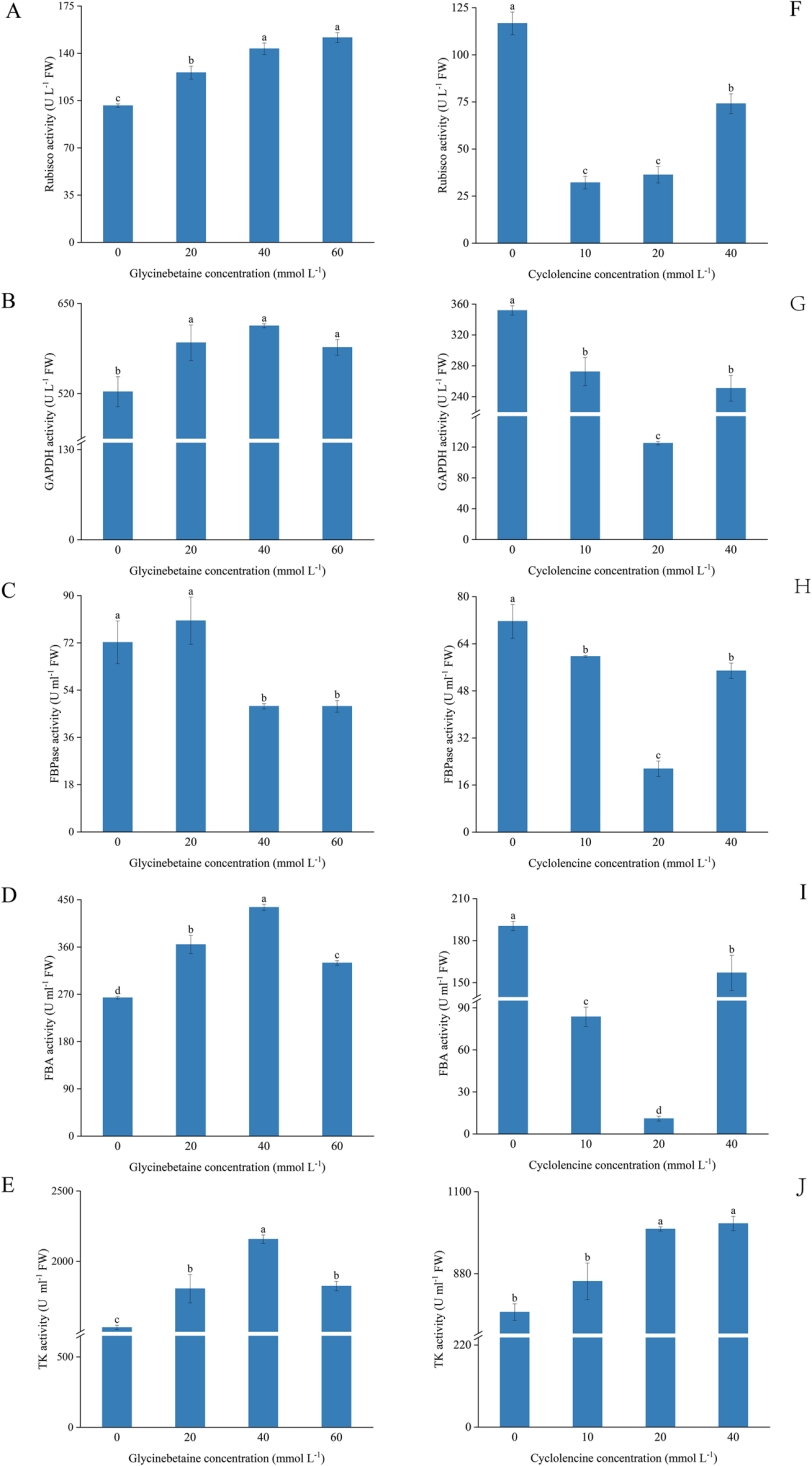
Effect of exogenous glycine betaine and cycloleucine on Calvin cycle enzyme activity in eggplant leaves. The different letters denote the significant difference among treatments (*p* < *0.05*), according to Duncan’s multiple tests. 0 mmol L^−1^: control.

RuBisCO, 3-glyceraldehyde-phosphate dehydrogenase (GAPDH), fructose-1,6-bisphosphate aldolase (FBA), and transketolase (TK) enzyme activities increased significantly (*p* < 0.05) after exogenous GB treatment, with the highest enzyme activity in the 40 mmol L^−1^ GB treatment (Fig. 4). The FBPase activity of plants treated with 20 mmol L^−1^ GB was higher than that of all other treatments (Fig. 4C). Exogenous cycloleucine treatment also significantly inhibited the activities of RuBisCO, GAPDH, FBPase, and FBA (Fig. 4F–I) but increased TK enzyme activity (Fig. 4J). Plants treated with 20 mmol L^−1^ cycloleucine had much lower GAPDH, FBPase, and FBA enzyme activities than those in the other treatments (Fig. 4G–I).

### Effect of exogenous glycine betaine and cycloleucine on chlorophyll fluorescence parameters in eggplant

**Figure 5:**
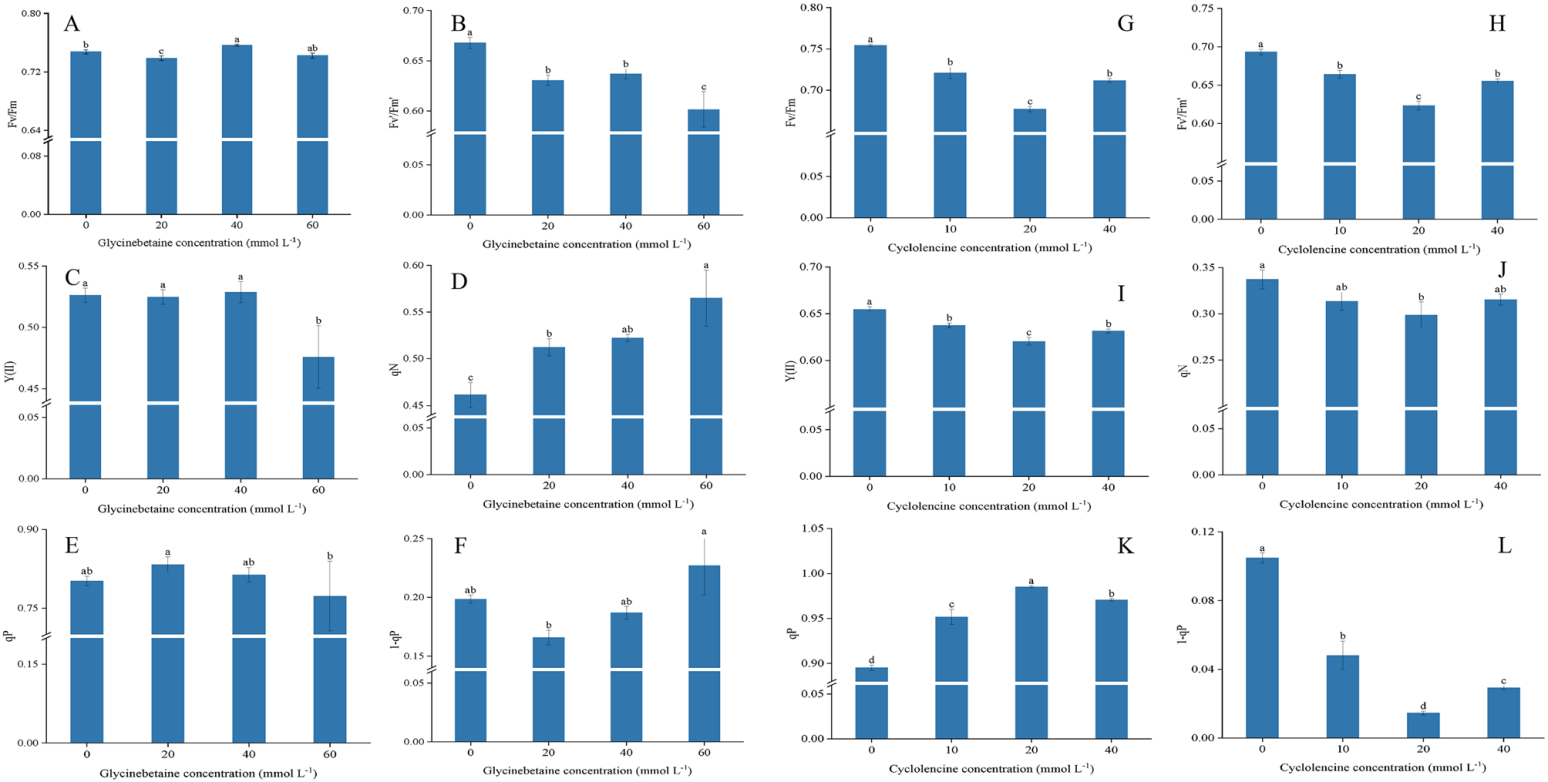
Effect of exogenous glycine betaine and cycloleucine on chlorophyll fluorescence parameters of eggplant leaves. The different letters denote the significant difference among treatments (*p* < *0.05*), according to Duncan’s multiple tests. 0 mmol L^−1^: control.

Fv'/Fm' decreased after spraying with exogenous betaine (Fig. 5B) and increased with 40 mmol L^−1^ GB treatment (Fig. 5A). However, 60 mmol L^−1^ betaine decreased the Y(II) values (Fig. 5C). The qN value increased with increasing GB concentration. qP increased after spraying with 20 mmol L^−1^ betaine, but 1-qP decreased. Thus, GB spraying can alter qN and qP in eggplant plants and increase heat dissipation, leading to a change in Y(II).

After spraying exogenous cycloleucine, Fv/Fm, Y(II), Fv'/Fm,' qN, and 1-qP decreased (Fig. 5G–J,L), but qP increased, with the most pronounced effect in plants treated with 20 mmol L^−1^ cycloleucine (Fig. 5). This suggests that cycloleucine may affect the ability to use the captured electron energy for photochemical reactions, causing changes in the PS II excitation pressure and affecting leaf fluorescence parameters.

### Effect of exogenous glycine betaine and cycloleucine on amino acids content

The 21 amino acids in eggplant treated with exogenous GB and cycloleucine are listed in Table 3. The top five amino acids in plants treated with exogenous GB were glutamate (143.26–335.10 ng g^−1^), aspartate (73.48–151.68 ng g^−1^), proline (33.96–130.13 ng g^−1^), serine (30.45–61.79 ng g^−1^), and alanine (43.88–52.63 ng g^−1^). Compared to 0 mmol L^−1^ (CK), glutamate, aspartate, serine, glutamine, lysine, valine, leucine, isoleucine, and methionine contents increased with increasing GB concentrations, whereas arginine, cystine, and glycine contents decreased (*p* < 0.05). In plants treated with 40 mmol L^−1^ GB, proline (130.13 ng g^−1^) and tryptophan (19.53 ng g^−1^) contents were significantly higher (*p* < 0.05) than the other treatments.

**Table 3:** Effect of exogenous glycine betaine and cycloleucine treatments on amino acid content of eggplant plants.

Amino acid (μg g^−1^)	Glycinebetaine				Cycloleucine			
	GB-0	GB-20	GB-40	GB-60	Cyc-0	Cyc-10	Cyc-20	Cyc-40
Lysine	28.71 ± 0.79d	37.26 ± 0.57c	50.36 ± 0.24b	53.69 ± 0.49a	46.26 ± 3.59c	92.7 ± 11.16b	150.94 ± 5.78a	148.8 ± 2.33a
Threonine	24.42 ± 0.95ab	23.33 ± 1.22b	28.35 ± 1.33a	23.32 ± 1.45b	90.1 ± 0.71bc	89.15 ± 0.83c	101.12 ± 1.76a	93.3 ± 1.13b
Phenylalanine	24.33 ± 3.45ab	19.41 ± 0.22b	27.59 ± 1.79a	20.83 ± 0.94ab	70.21 ± 2.00b	76.56 ± 1.36ab	82.67 ± 1.23ab	54.24 ± 3.85b
Tryptophan	7.69 ± 0.35c	8.34 ± 0.42c	19.53 ± 0.06a	9.79 ± 0.13b	130.32 ± 4.46a	62.00 ± 2.78b	27.06 ± 0.98c	31.24 ± 0.42c
Leucine	8.54 ± 0.35b	9.24 ± 0.63b	10.67 ± 0.31ab	11.94 ± 1.03a	13.67 ± 0.15c	15.2 ± 0.52c	36.95 ± 0.50b	63.14 ± 2.55a
Isoleucine	9.73 ± 0.09d	10.84 ± 0.06c	12.64 ± 0.58b	14.29 ± 0.06a	14.55 ± 0.26c	16.83 ± 1.17c	40.32 ± 2.28b	64.26 ± 2.78a
Valine	13.86 ± 0.26d	16.79 ± 0.48c	19.97 ± 0.35b	23.69 ± 0.42a	49.21 ± 5.20a	26.24 ± 1.54b	44.07 ± 2.16a	47.63 ± 4.15a
Methionine	4.76 ± 0.19b	5.35 ± 0.27b	5.25 ± 0.20b	7.58 ± 0.05a	8.40 ± 0.07c	9.43 ± 0.41c	28.99 ± 1.48b	45.91 ± 2.48a
Cysteine	6.38 ± 0.25a	6.16 ± 0.10a	6.03 ± 0.03a	6.04 ± 0.07a	5.98 ± 0.03a	6.04 ± 0.08a	6.12 ± 0.13a	6.18 ± 0.21a
Alanine	43.88 ± 1.40a	50.49 ± 3.20a	52.63 ± 3.38a	47.76 ± 4.02a	101.86 ± 0.25b	70.72 ± 2.99c	113.7 ± 2.66ab	121.73 ± 9.16a
Glycine	5.84 ± 0.62a	3.55 ± 0.16b	4.34 ± 0.42b	4.65 ± 0.16ab	3.80 ± 0.17d	13.6 ± 0.23c	51.94 ± 0.61a	32.61 ± 0.04b
Tyrosine	0.48 ± 0.05d	4.70 ± 0.34a	3.63 ± 0.10b	2.96 ± 0.13c	11.49 ± 0.17c	20.65 ± 0.11b	27.12 ± 2.18a	28.00 ± 1.96a
Aspartate	73.48 ± 0.97b	151.68 ± 2.98a	144.30 ± 2.94a	151.05 ± 2.68a	491.01 ± 15.51a	267.59 ± 1.65b	434.47 ± 29.03a	469.3 ± 25.62a
Proline	53.29 ± 1.79c	69.74 ± 1.12b	130.13 ± 0.55a	33.96 ± 1.08d	746.30 ± 8.88a	476.39 ± 14.5c	698.68 ± 37.85b	344.13 ± 11.98d
Serine	30.45 ± 2.27b	55.96 ± 1.56a	54.38 ± 1.68a	61.79 ± 5.06a	142.35 ± 7.31c	148.95 ± 7.86c	273.42 ± 11.47b	313.2 ± 8.41a
Glutamate	143.26 ± 3.66c	312.29 ± 8.52a	258.39 ± 11.26b	335.1 ± 3.82a	503.01 ± 6.43d	671.15 ± 9.22b	788.13 ± 15.03a	565.37 ± 13.86c
Glutamine	30.55 ± 0.84d	40.74 ± 1.23c	48.83 ± 3.27b	55.26 ± 0.40a	47.82 ± 3.55c	82.78 ± 3.21b	163.26 ± 11.88a	150.67 ± 2.68a
Arginine	32.10 ± 0.19a	20.93 ± 0.28b	21.28 ± 0.95b	31.04 ± 2.24a	24.36 ± 1.34d	91.37 ± 0.86c	300.59 ± 17.38a	193.38 ± 1.89b
Asparagine	7.50 ± 0.19b	19.19 ± 0.54a	7.59 ± 0.19b	7.75 ± 0.13b	132.93 ± 3.96a	76.05 ± 1.65c	104.24 ± 0.23b	100.58 ± 1.64b
Histidine	12.94 ± 0.21b	9.89 ± 0.34c	9.02 ± 0.32c	16.37 ± 0.75a	29.35 ± 3.14d	71.77 ± 3.53c	82.72 ± 1.72b	193.67 ± 1.41a
Cystine	5.32 ± 0.54a	5.12 ± 0.40ab	4.09 ± 0.18b	4.69 ± 0.02ab	4.02 ± 0.06b	5.89 ± 0.11a	3.84 ± 0.10b	5.56 ± 0.45a

The growth parameters of eggplant plants were obtained on the 14th day after the end of the treatment. The results are shown as the mean ± SE of three replicates, and the different letters denote significant differences between treatments (*p* < 0.05), according to Duncan's multiple range tests. CK: 0 mmol L^−1^; GB-20:20 mmol L^−1^ glycine betaine; GB-40:40 mmol L^−1^ glycine betaine; GB-60: 60 mmol L^−1^ glycine betaine. Cyc-0: 0 mmol L^−1^; Cyc-10: 10 mmol L^−1^ cycloleucine; Cyc- 20: 20 mmol L^−1^ cycloleucine; Cyc-40: 40 mmol L^−1^ cycloleucine.

The amino acid contents of plants treated with exogenous cycloleucine were glutamate > proline > aspartate > serine > arginine > glutamine > lysine > asparagine > alanine > histidine > threonine > phenylalanine > tryptophan > valine > isoleucine > leucine > glycine > methionine > tyrosine > cysteine. The glutamate, proline, arginine, threonine, and glycine contents of plants treated with 20 mmol L^−1^ cycloleucine were higher than those of the other treatments (*p* < 0.05)*.* The methionine content of cycloleucine-treated plants significantly increased with increasing cycloleucine concentrations.

### Effect of exogenous glycine betaine and cycloleucine on endogenous hormone content

**Figure 6:**
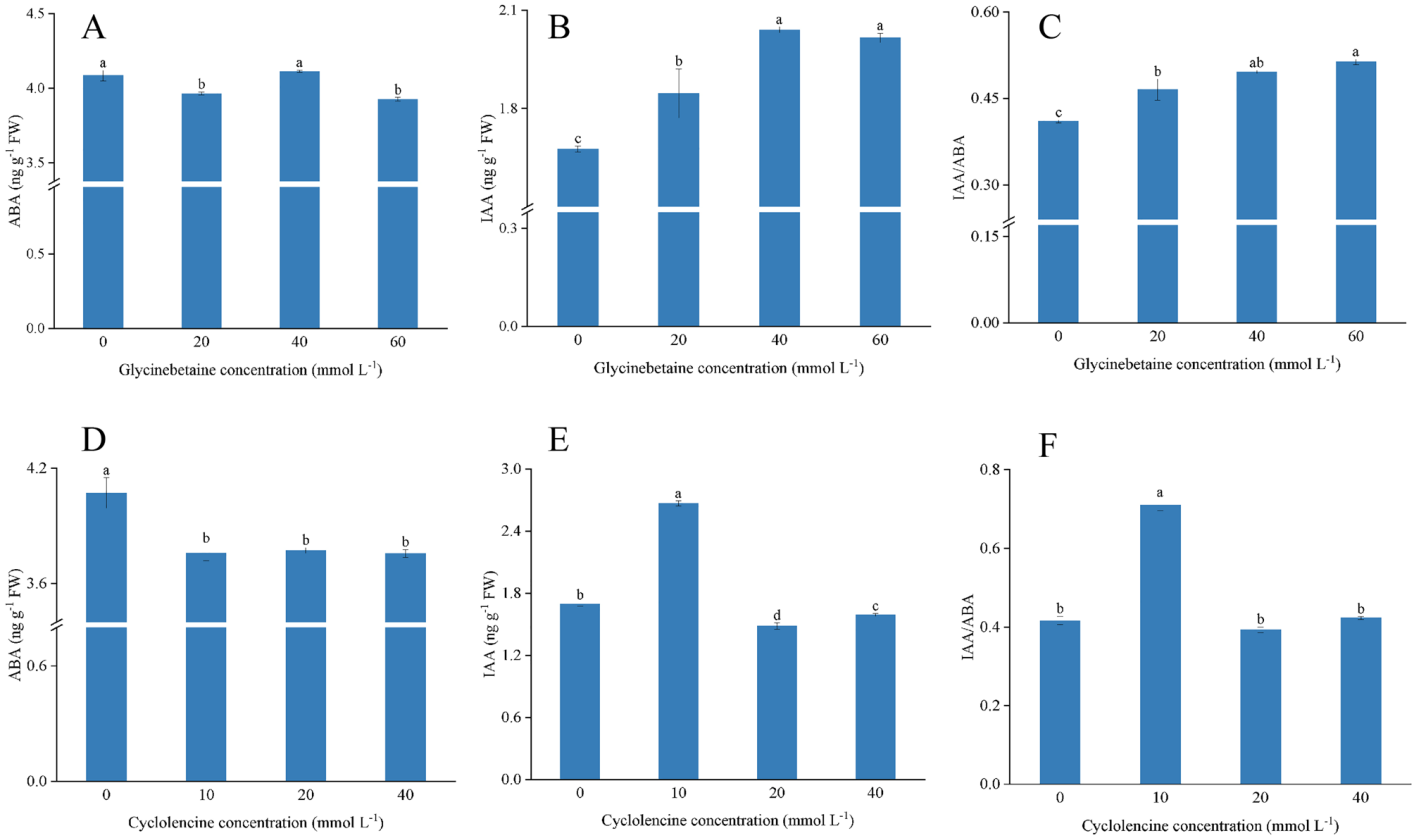
Effect of exogenous glycine betaine and cycloleucine on endogenous hormone content. The different letters denote the significant difference among treatments (*p* < *0.05*), according to Duncan’s multiple tests. 0 mmol L^−1^: control.

Plants treated with 40 mmol L^−1^ GB had the highest ABA content (4.11 ng g^−1^ FW), which increased by 3.79% and 4.58% (*p* < 0.05) compared with the 20 mmol L^−1^ and 60 mmol L^−1^ treatments, respectively (Fig. 6A). The IAA content increased with increasing betaine concentration (Fig. 6B). Compared with 0 mmol L^−1^, the IAA content of plants treated with 20, 40, and 60 mmol L^−1^ increased by 10.12%, 21.43%, and 20.24%, respectively (*p* < 0.05). The IAA/ABA ratio also increased with increasing betaine concentration (Fig. 6C).

The ABA content of plants sprayed with cycloleucine was significantly lower than that of plants sprayed with 0 mmol L^−1^; however, the differences between the 10, 20, and 40 mmol L^−1^ treatments were insignificant. Plants treated with 10 mmol L^−1^ cycloleucine had the highest IAA content (2.67 ng g^−1^ FW), and those treated with 20 mmol L^−1^ cycloleucine had the lowest IAA content. The plants treated with 10 mmol L^−1^ had the highest IAA/ABA value (Fig. 6F), significantly higher than the other treatments (*p* < 0.05).

## Discussion

GB has been widely used to alleviate abiotic stress in plants and has shown good results^19,21^. GB mitigates abiotic stresses mainly by protecting cell membranes^19^, maintaining normal intracellular osmotic pressure^20^, reducing photosynthetic system damage, and stabilizing related enzyme activities^23^. We found that spraying exogenous GB on eggplant seedlings under normal conditions promoted plant growth, facilitated plant development, and improved photosynthesis in eggplants. In our study, the plant height, shoot fresh weight, and shoot dry weight of eggplant increased significantly after exogenous spraying with different GB concentrations, with 20 and 40 mmol L^−1^ being the most effective. It has been shown that when GB is applied exogenously, it is absorbed and quickly moves from the leaves to meristematic tissues, such as buds and stem tips, thus promoting flowering and growth^37,38^. Regarding plant appearance and morphology, 40 mmol L^−1^ GB significantly increased the flowering time of plants (Fig. 1A). However, 60 mmol L^−1^ betaine had some inhibitory effect on plant growth (Fig. 1A); plant height, stem thickness, root fresh weight, and root dry weight were slightly lower with 0 mmol L^−1^ (control). This indicates that exogenous GB can promote growth at low concentrations under normal conditions^25,39^.

Conversely, the plant height, stem diameter, shoot fresh weight, and root fresh weight of eggplant plants treated with different concentrations of exogenous cycloleucine decreased significantly with an increase in cycloleuvine concentration compared with 0 mmol L^−1^. These results indicated that the exogenous spraying of cycloleucine significantly inhibited the growth and development of eggplant plants. In terms of the plant phenotype, 10 mmo L^−1^ cycloleucine delayed the flowering time (Fig. S1), 20 mmol L^−1^ and 40 mmol L^−1^ cycloleucine directly caused stunted plant growth and development, and even led to the loss of apical dominance and an increase in the number of lateral shoots (Figs. S1, S2). These results are similar to those of Colombarin^32^ on potatoes, indicating that plant growth and development were inhibited after cycloleucine application.

Epidermal hairs on plant leaves can effectively reduce leaf water gain^40^, protect leaf flesh cells from damage^41^, and reduce pathogenic fungal attacks^42^. I In this experiment, the density of abaxial hairs on eggplant leaves increased significantly with increasing GB concentrations compared to the control treatment, and the morphology was sharper. However, treatment with cycloleucine spray resulted in a significant morphological change in eggplant leaf abaxial hair, which appeared flattened, and the number of individual hair-like branches decreased. In terms of magnification, 40 × magnification was required to observe leaf abaxial hairs of control plants and plants sprayed with GB, while 110 × magnification was required for cycloleucine-treated plants. This suggests that the morphology of abaxial hairs of leaves may be smaller after cycloleucine treatment.

Previous studies have shown that exogenous GB can significantly improve plant gas exchange properties^43^, stabilize photosynthetic pigments and chlorophyll fluorescence, and promote plant growth^25^. In this study, Pn and Gs increased and then decreased with increasing betaine concentration. It also validated the improved effect of GB on plant gas exchange parameters and indicated that the main factor of photosynthetic limitation could be the stomatal limitation^24,39^. Plants treated with 20 and 40 mmol L^−1^ GB showed a significant increase in Pn. In addition, according to Gs and Ci, the stomatal opening of leaves was significantly increased after betaine spraying, while Tr was the lowest in plants treated with GB at 20 mmol L^−1^ concentration and even significantly lower than CK. Combined with Pn, Gs and Ci, the main reason could be due to a decrease in the number of normally open stomata on the leaves, resulting in a decrease in Tr^44^.

Cycloleucine acts as a methylation inhibitor and affects photosynthesis in plants by inhibiting S-adenosylmethionine synthase and, thus, RNA methylation^45^. Gas exchange parameters were affected in plants sprayed with cycloleucine. Compared with the control plants, Pn, Gs, and Tr were reduced in cycloleucine-treated plants (Fig. 3E,F,H), whereas Ci levels were elevated. This suggests that the effect of cycloleucine on the gas exchange parameters may be achieved by affecting the uptake and utilization of CO_2_. This is because, as the concentration of cycloleucine increased, a rising and then decreasing trend was observed in Pn, and Gs, Ci, and Tr showed an increasing trend. This result suggests that cycloleucine may not affect the gas exchange capacity but rather the assimilation capacity of the photosynthetic system for CO_2_.

Key enzymes of the Calvin cycle (RuBisCO, GAPDH, FBA, FBPase, and TK) play an irreplaceable role in photosynthesis^46,47^. RuBisCO and FBPase play important roles in carbon fixation: reduction and RuBP regeneration, respectively. FBA and TK promote CO_2_ assimilation in leaves and carbon flow in the Calvin cycle, and GAPDH is a key enzyme in glycolysis^47^. The exogenous administration of GB has been shown to elevate the activity of key Calvin cycle enzymes, which is similar to our results^48,49^. We showed that exogenous spraying of GB increased RuBisCO, GAPDH, FBA, FBPase, and TK activity, whereas plants sprayed with cycloleucine showed decreased RuBisCO, GAPDH, FBA, FBPase, and TK enzyme activity. This suggests that GB can enhance the key activities of the Calvin cycle under normal conditions. Meanwhile, plants sprayed with cycloleucine had decreased activity of key Calvin cycle enzymes, which may be due to the effect of cycloleucine on RNA methylation^32,50^, affecting enzyme synthesis and transport, resulting in decreased enzyme activity. Compared to the control treatment, GB increased chlorophyll content (Table 2)^38,51^. The leaves of cycloleucine-treated plants turned yellow, and the chlorophyll content decreased. This indicates that cycloleucine may increase the rate of chlorophyll degradation and decrease the synthesis rate^32,51^.

Chlorophyll fluorescence provides insights into the cellular response to exogenous substances. It can quantify changes in the donor and acceptor sides of PSII reaction centers, thus providing information about energy uptake, utilization, and transport^52^. Compared to the control, GB treatment significantly increased Fv/Fm, qN, and qP (Fig. 5A,D,E) and significantly decreased Fv′/Fm′ (Fig. 5B). Exogenous GB affects Fv/Fm mainly by promoting electron transport from Q_A_ to Q_B_ in vesicle-like membranes^53,54^. Our results showed that exogenous GB elevated plant Fv/Fm (Fig. 5A). However, Fv'/Fm' decreased, indicating that spraying 40 mmol L^−1^ GB significantly promoted Fv/Fm compared to CK (*p* < 0.05), a result consistent with the findings in tomatoes^54^.

Similarly, qN increased because of dynamic photoinhibition (Fig. 5D). As qN increased, qP decreased (Fig. 5D,E), indicating a reduction in the actual photochemical rate of PSII^55^. Also, 1-qP decreased in plants treated with 20 and 40 mmol L^−1^ GB, indicating that GB could reduce the excitation stress faced by PSII, improve the efficiency of excitation energy capture (Fig. 5B), and inhibit the reduction in Y(II) (Fig. 5C). In contrast, cycloleucine was responsible for a significant decrease in Fv/Fm, Fv'/Fm,' Y(II), qN, and 1-qP (Fig. 5G–J,L), and a significant increase in qP (Fig. 5K). This indicates that chronic and dynamic photoinhibition are affected by cycloleucine spraying^46,47^. qN decreased while qP was enhanced, indicating that cycloleucine reduced heat dissipation and enhanced photosynthesis. Plants treated with 20 mmol L^−1^ cycloleucine showed increased Pn (Fig. 3E), which also verifies this result. The 1-qP value decreased significantly (Fig. 5L), also indicating that cycloleucine affected the PS II excitation pressure.

Amino acids are the basic units for synthesizing proteins and other metabolites. They are commonly involved in physiological and biochemical processes in plants, such as the regulation of osmotic pressure^56^, alteration of enzyme activity, regulation of stomatal opening, and ion transport^57^. Glutamate can serve as a carbon pool for δ-aminolevulinic acid during chlorophyll synthesis^58^, and δ-aminolevulinic acid is formed when glutamate reacts with tRNA, indicating the importance of glutamate in chlorophyll biosynthesis^59^. In this experiment, both betaine and cycloleucine applications significantly increased the glutamate content of the plants compared to that of the control (Table 3). GB spraying increased the chlorophyll content, verifying that exogenous GB elevates glutamate content^60^.

In contrast, treatment with cycloleucine increased glutamate content but decreased chlorophyll content. This suggests that spraying with cycloleucine may have inhibited glutamate utilization by the plants, leading to its formation and accumulation. Serine plays an active role in plant metabolic activities such as phospholipid biosynthesis, photorespiration, and biosynthesis of other amino acids^61^. The serine content of the plants sprayed with exogenous GB was significantly higher than that of the controls^62^. Meanwhile, the differences between plants treated with 20, 40, and 60 mmol L^−1^ GB were insignificant, indicating that the spraying of exogenous GB may have promoted serine synthesis. However, other reasons limited the increase in serine content.

Glycine is also a precursor of GB, which can be oxidized to form serine^61,63^. The increase in serine content and the decrease in glycine content after spraying exogenous GB may be due to the fact that exogenous GB inhibited glycine synthesis and promoted the conversion of glycine to serine. The glycine content in cycloleucine treatment increased and then decreased, whereas serine content increased. This indicates that the metabolic synthesis pathways of both serine and glycine were influenced by cycloleucine. Valine, leucine, and isoleucine can act as alternative electron donors in the mitochondrial electron transport chain^64,65^. After spraying exogenous GB, valine, leucine, and isoleucine levels increased with increasing GB concentration. The corresponding photosynthetic and chlorophyll fluorescence parameters improved^44^, indicating that GB promoted the synthesis and utilization of valine, leucine, and isoleucine^66^. However, photosynthetic and chlorophyll fluorescence parameters decreased with cycloleucine treatment, but leucine and isoleucine content increased, while valine content decreased. This suggests that cycloleucine may have inhibited the utilization of leucine and isoleucine but suppressed valine synthesis. Aspartate is a key amino acid for plant growth, development, and adaptation to environmental challenges, most likely through its interaction with plant hormones, such as growth hormones and ethylene^67^. The change in the aspartate content of both exogenous GB and cycloleucine-treated plants was inversely proportional to the IAA content (Fig. 6B,E; Table 3), which also verified the above conclusion^68^.

Aspartate, glutamate, alanine, and glycine support flowering, whereas threonine and phenylalanine inhibit it^57,69^. Combined with the plant phenotypes (Fig. 1A), aspartate, glycine, alanine, threonine, and phenylalanine contents were significantly increased after GB spraying compared to the control. However, flowering occurred earlier in plants treated with GB at 40 mmol L^−1^. This indicates that, although GB increases amino acid content, it also inhibits the action of threonine and phenylalanine, causing their formation and accumulation. Aspartic acid, glycine, alanine, threonine, and phenylalanine in cycloleucine-treated plants increased with increasing cycloleucine concentrations. However, spraying with cycloleucine significantly affected the reproductive growth of the plants (Fig. 1B, Fig. S1), indicating that as the plants took up cycloleucine, it directly affected the conversion and utilization of aspartic acid, glycine, alanine, threonine, and phenylalanine, causing their accumulation.

Methionine and threonine are precursors of isoleucine^70,71^. This conclusion is also verified by the fact that the trends of methionine, threonine, and isoleucine are the same regardless of whether GB or cycloleucine is sprayed. As for tryptophan, a synthetic precursor of IAA^72^, the trends of tryptophan and IAA contents in the plants were consistent after GB application. However, the IAA and tryptophan contents of plants sprayed with cycloleucine decreased. This indicates that cycloleucine affects tryptophan synthesis. Methionine can be used in cellular metabolism as a biomodulatory element for mRNA initiation, translation, protein composition, and S-adenosylmethionine (SAM)^71,73^. The high concentration of betaine used in this experiment catalyzed the increase in methionine content^74^.

In contrast, methionine content increased with increasing cycloleucine concentration, which worsened plant growth. This suggests that the effect of cycloleucine on methionine content may be consistent with the conversion and utilization of methionine in the metabolic pathway rather than the inhibition of methionine synthesis. Photorespiration is the main source of serine and glycine amino acids in photosynthetic tissues^75,76^. The increased serine and glycine content in plants sprayed with cycloleucine indicates enhanced photorespiration and increased energy loss in their leaves^77,78^.

Endogenous hormones play important roles in plant growth and development^79,80^. They participate in various physiological and biochemical processes in plant cells, tissues, and organs^81^, directly affecting plant growth and development^82^. GB treatment changes the hormone metabolic pathways in plants and affects the hormone content^83^. In this study, the leaf ABA content decreased (Fig. 6A), but the IAA content increased (Fig. 6B) after spraying with the exogenous GB. The ABA content in leaves affects the opening and closing of stomata, which in turn affects photosynthesis in the leaves^84^. After a decrease in ABA content (Fig. 6A), there was an increase in both the Pn and Gs (Fig. 3A,B), similar to the findings of Chen et al.^85^.

IAA promotes cell growth and differentiation^86^ and root development and plant growth^87,88^. After spraying exogenous GB, the IAA content increased and directly promoted plant growth and development (Fig. 1A), with the best effect observed for GB at 40 mmol L^−1^. After spraying cycloleucine, the ABA content decreased (Fig. 6D), but the IAA content increased and then decreased (Fig. 6E). This indicated that the endogenous hormone level of the plant decreased, and normal plant growth and development were inhibited (Fig. 1B)^89,90^. Changes in the hormone levels also resulted in delayed floral bud differentiation and flowering^91,92^.

In conclusion, the spraying of exogenous GB promoted the growth of eggplant seedlings, increased the fresh and dry weights of the plants, promoted the development of abaxial leaf hairs, promoted the differentiation of flower buds, shortened the flowering time, improved the photosynthetic and fluorescence parameters in eggplant plants, and increased the amino acid content, with the greatest effect at 40 mmol L^−1^ concentration of GB. Spraying exogenous cycloleucine at 20 mmol L^−1^ significantly inhibited the growth and development of eggplant, leading to a decrease in the gas exchange parameters of plant leaves, inhibiting the development of abaxial leaf hairs, leading to a decrease in phytohormone content, and affecting the conversion and utilization of amino acids in the plant.

## Methods

### Plant material and experimental treatments

The experiment was conducted from May 2021 to March 2022 in an artificial climate chamber (RDN-400E-4, Ningbo, Zhejiang) at the College of Horticulture, Gansu Agricultural University (36°05′ 39.86″ N, 103°42′ 31.09″ E). The test eggplant variety was 'Xingniang' (long eggplant, the main cultivar in Gansu Province), purchased from Jiuquan Xiahe Seed Co., Ltd. GB (purity > 99%, CAS: 107-43-7) and cycloleucine (purity > 98%, CAS: 52-52-8) were purchased from Shanghai Yuanye Bio-Technology Co., Ltd.

The uniform-sized and full-textured eggplant seeds were selected, soaked in warm water at 55 °C for 30 min, and then put in germinating box at (28 ± 1) °C (day)/(18 ± 1) °C (night). After 96 h, germinated seeds were sown in plastic pots (10 cm × 10 cm) with a cultivation substrate of grass charcoal: vermiculite: perlite = 3:1:1. After germination, the seedlings were incubated in a climatic chamber at (28 ± 1) °C (day)/(18 ± 1) °C (night), with a light intensity of 30,000 lx. When the seedlings grew to two leaves, they were watered with ½ Hoagland's nutrient solution and water at intervals; when they grew to four leaves, they were watered with Hoagland's full nutrient solution and water at intervals. Before starting the spraying of exogenous substances, the weaker plants in each pot were removed, leaving only the better-growing plants. Uniformly sized plants were treated with different concentrations of exogenous GB and cycloleucine foliar sprays.

The experiment was conducted using a completely randomized design with three replicates. Three different betaine concentrations were used: 20, 40, and 60 mmol L^−1^, with 0 mmol L^−1^ as the control treatment. Three cycloleucine concentrations were also tested: 10, 20, and 40 mmol L^−1^, with 0 mmol L^−1^ as the control treatment. All treatments lasted for 3 days. The culture conditions at the end of treatment were the same as before. The growth physiology of 60-day-old seedlings (14 days after GB and cycloleucine treatment) was measured and analyzed using the indicators described below.

This study complies with local and national guidelines. Plant experiments were also performed in accordance with the relevant guidelines and regulations. As *Solanum melongena L.* is a commonly grown vegetable, no permission is required to collect it.

### Growth index determination

Nine pots from each treatment were randomly selected to measure the plant height and stem diameter. Then, the plants were divided into shoot and root parts, the root substrate was rinsed, and the surface water was drained. After measuring the fresh weight of aboveground and belowground parts, they were placed in an oven at 105 °C for 30 min and then dried at 75 °C until constant weight^93^. The dried plants were crushed and ground for subsequent determination of other indices. All the remaining plants of each treatment were immediately frozen in liquid nitrogen and stored at − 80 °C in a refrigerator to determine other indexes.

### Measurement of photosynthetic pigments, photosynthetic and gas exchange parameters, and chlorophyll fluorescence parameters

Random samples were collected from each treatment of eggplant plants to determine leaf chlorophyll content. Using the method of Wang, et al.^94^ and Parvaiz Ahmad et al.^95^: samples were collected with a 0.5 cm diameter leaf perforator, 0.1 g of leaf samples were accurately weighed, placed in a 20 mL stoppered test tube, 10 mL of 80% acetone was added, mixed thoroughly, sealed with paraffin film, and placed in a dark environment for 48 h to extract chlorophyll until the leaves turned white. The OD values of the extracts were measured at 663 nm and 645 nm using a UV-1780 UV spectrophotometer (Shimadzu Instruments (Suzhou) Co., Ltd., Suzhou, China). Chlorophyll content was calculated according to Arnon's^96^ method using the following equations:

$$Chl. \, a \, (mg \, {g}^{-1} \, FW)=\frac{12.7\times \mathrm{OD}663-2.59\times \mathrm{OD}645}{\mathrm{V}/1000\mathrm{m}}$$

$$Chl. \, b \, (mg \, {g}^{-1} \, FW)=\frac{22.8\times \mathrm{OD}645-4.67\times \mathrm{OD}663}{\mathrm{V}/1000\mathrm{m}}$$

$$Chl .\, T \, (mg \, {g}^{-1} \, FW)=\frac{22.9\times \mathrm{OD}645+8.04\times \mathrm{OD}663}{\mathrm{V}/1000\mathrm{m}}$$

V in the equation represents the total volume of the extraction solution, and m represents the sample mass.

When plants were grown for up to 60 days (day 14 after treatment), gas exchange parameters, including Pn, Ci, Gs, and Tr, were measured using a portable photosynthesis system (CIRAS-2, PP system). The second leaf of five plants was selected for a replicate of each treatment. The conditions set during measurement were as follows: leaf area, 1.7 cm^2^; chamber flow rate, 200 mL min^−1^; photosynthetically active irradiation, 1000 µmol m^−2^ s^−1^; carbon dioxide (CO_2_) concentration, 400 µmol mol^−1^; air temperature, 25 °C; and relative humidity, 75%^46^.

Three seedlings were randomly selected for each treatment to measure chlorophyll fluorescence parameters. After 30 min of dark acclimation, the third fully expanded functional leaf was cut and placed on a fluorimeter measuring table. Then, chlorophyll was measured using a modulated chlorophyll fluorescence imager (IMAGING-PAM; Heinz WaIz GmBH, Effeltrich, Germany). The parameters were set to a measurement light illumination intensity of 0.1 μmol m^−2^ s^−1^, photochemical light intensity of 81 μmol m^−2^ s^−1^, saturating pulsed light intensity of 2700 μmol m^−2^ s^−1^, and pulsed light time of 0.8 s. Saturating pulsed light was hit every 20 s for a total of 15 hits^47^. The initial fluorescence Fo and maximum fluorescence Fm in the dark were obtained by hitting the saturation pulse light, the maximum photochemical efficiency of PSII was calculated, and the steady-state fluorescence Fs was obtained under photochemical light after 300 s of continuous supply of photochemical light. Meanwhile, the maximum fluorescence yield Fm′ under light was obtained after 0.8 s of the saturation pulse light, and the actual photochemical efficiency Y(II) and photochemical burst coefficient qP were obtained^97,98^.

$$\frac{Fv}{Fm}=\frac{Fm-Fo}{Fm}$$

$$Y\left(II\right)=\frac{F{^{\prime}}m-Fs}{F{^{\prime}}m}$$

$$qP=\frac{Fm{^{\prime}}-Fs}{Fm{^{\prime}}-Fo{^{\prime}}}$$

$$qN=\frac{Fm-Fm{^{\prime}}}{Fm-Fo}$$

$$\frac{Fv{^{\prime}}}{Fm{^{\prime}}}=\frac{(\mathrm{Fm{^{\prime}}}-\mathrm{Fo{^{\prime}}})}{Fm{^{\prime}}}$$

### Morphological observation of leaf abaxial hairs

When plants were grown for 60 days, the middle part of their leaves was cut off in 5 × 5 mm pieces with a scalpel and immersed in 2.5% glutaraldehyde fixative for electron microscopy (Shanghai Yuanye Bio-Technology Co., Ltd., Shanghai, China). The dehydration method described by Yang, et al.^46^ was used. The fixative containing the samples was refrigerated at 4 °C for 24 h, washed four times with phosphate buffer (0.1 M PBS, pH 7.4) at 15 min intervals, and then dehydrated with ethanol (30, 40, 50, 60, 65, 70, 75, 80, 85, 90, 95%) in steps of 20 min each. Then, it was transferred to 100% ethanol for three immersion washes, 30 min each. In the end, the ethanol was replaced with tert-butanol (30, 50, 70, 80, 85, 90, 95, 100, 100, 100%). After drying, the samples were sprayed with gold using a gold sputter (Magnetron Sputter, MSP-1S; Vacuum Device, Tokyo, Japan), and the morphology of the leaf abaxial hairs was observed and photographed using a scanning electron microscope (SEM, S-3400N, Hitachi, Tokyo, Japan).

### Calvin cycle key enzymes

The key enzymes of the Calvin cycle include RuBisCO, FBPase, FBA, GAPDH, and TK. The activities of these five enzymes were measured (three eggplant plants per treatment, n = 3) using an ELISA kit (Yaji Biotech, Shanghai, China). Leaf samples were completely ground with 0.05 mM Tris–HCl and 0.1 M phosphate buffer (pH 7.4) and centrifuged (4 °C, 3000×*g*, 15 min), and the supernatant was used to determine the enzyme activity. This method was performed according to the manufacturer's instructions. The amount of enzyme required to convert 1 µmol of the substrate in 1 min is called enzyme activity (U). U L^−1^ is the international unit of enzyme activity (U L^−1^ stands for enzyme activity per liter of enzyme preparation; U mL^−1^ stands for enzyme activity per mL of enzyme preparation).

### Determination of endogenous hormones and amino acid content

For the determination of IAA and ABA content, 0.5 g of sample tissue was rapidly ground into powder using liquid nitrogen and packed into a 10 mL centrifuge tube with 5 mL of extraction solution (n-propanol: distilled water: hydrochloric acid = 2:1:0.002, V: V) and shaken for 30 min at 4 °C on a shaker at 100 rpm. The tube was removed, 2 mL of dichloromethane was added, and the mixture was shaken again for 30 min. Centrifuged in a refrigerated centrifuge (4 °C, 13,000 rpm, 5 min) after completion. The oily liquid was aspirated in a lyophilized bottle and stored at – 80 °C for 14 h before drying with a freeze dryer. The finished product was a white powder (it takes 24 h to obtain the finished product). Then, it was re-dissolved in 80% methanol aqueous solution, filtered through 0.22 μm organic membrane, and detected on the machine. Endogenous IAA and ABA contents were determined using high-performance liquid chromatography (Agilent 1100 series; Agilent Technologies, Santa Clara, CA, USA) with 10 μL of injected sample volume. A C18 inverse-phase column (ZORBAX SB-C18, 4.6 × 250 mm, 5 μm) was used at 30 °C with the mobile phase in acetonitrile: methanol: 0.6% acetic acid solution (5:50:45, V: V: V) at a flow rate of 1.0 mL min^−1^ and wavelengths of 218 nm (IAA) and 262 nm (ABA). The content of IAA and ABA was determined according to the method defined by Heidari et al.^99^.

An ultra-high liquid chromatography-mass spectrometry system (UPLC-MS, Agilent 1290-6460, LC/MS, Agilent Technologies) was used to determine the content of 21 free amino acids^94^. The method was as follows: 100 mg of fresh samples of each species were accurately weighed and extracted with 0.5 mol L^−1^ hydrochloric acid aqueous solution (1 mL). The solution was mixed by vortexing for 5 min, sonicated in a water bath at 25 °C for 20 min, and then centrifuged at 20,000×*g* for 20 min. Then, 250 µL of the supernatant was transferred to a liquid chromatography vial, diluted to 1 mL with 80% aqueous acetonitrile, and passed through an organic phase microporous membrane of 0.22 μm for determination.

The injection volume was 1 μL, and the column used was Agilent Infinity Lab Poroshell 120 HILIC-Z (2.1 × 100 mm). The mobile phase A was 20 mmol L^−1^ ammonium formate aqueous solution (pH = 3), mobile phase B was 20 mmol L^−1^ ammonium formate aqueous solution (pH = 3) and 90% acetonitrile aqueous solution V:V = 9:1. The mass spectrometry conditions were in ESI positive ionization mode with a desiccator temperature of 330 °C, a gas flow rate of 13.0 L min^−1^, an intrathecal gas temperature of 390 °C, an intrathecal gas flow rate of 12.0 L min^−1^, and capillary voltage of 1500 V.

### Statistical analysis

All experimental data were analyzed using IBM Statistical Product and Service Solutions (SPSS) Statistics version 22.0 (IBM Corp., Armonk, NY, USA), and the statistical significance of treatment means was evaluated using Duncan's multiple range test (*p* < 0.05). All data are presented as mean ± SE. Data figures, correlation analysis, and principal component analysis were generated using Origin Pro 2021.

## Supplementary Information

Supplementary Figure S1.

Supplementary Figure S2.

## Data Availability

The datasets generated during and/or analysed during the current study are available from the corresponding author on reasonable request. This study complies with local and national guidelines. Plant experiments were also performed in accordance with the relevant guidelines and regulations.
