# Supplementary material for: Effects of exogenous glycine betaine and cycloleucine on photosynthetic capacity, amino acid composition, and hormone metabolism in Solanum melongena L
Source: Sci Rep. 2023 May 10;13:7626. doi: 10.1038/s41598-023-34509-w (PMC10172174; doi:10.1038/s41598-023-34509-w)
Supplement: Supplementary file 1 — Supplementary Figure S1. [file 41598_2023_34509_MOESM1_ESM.pdf]

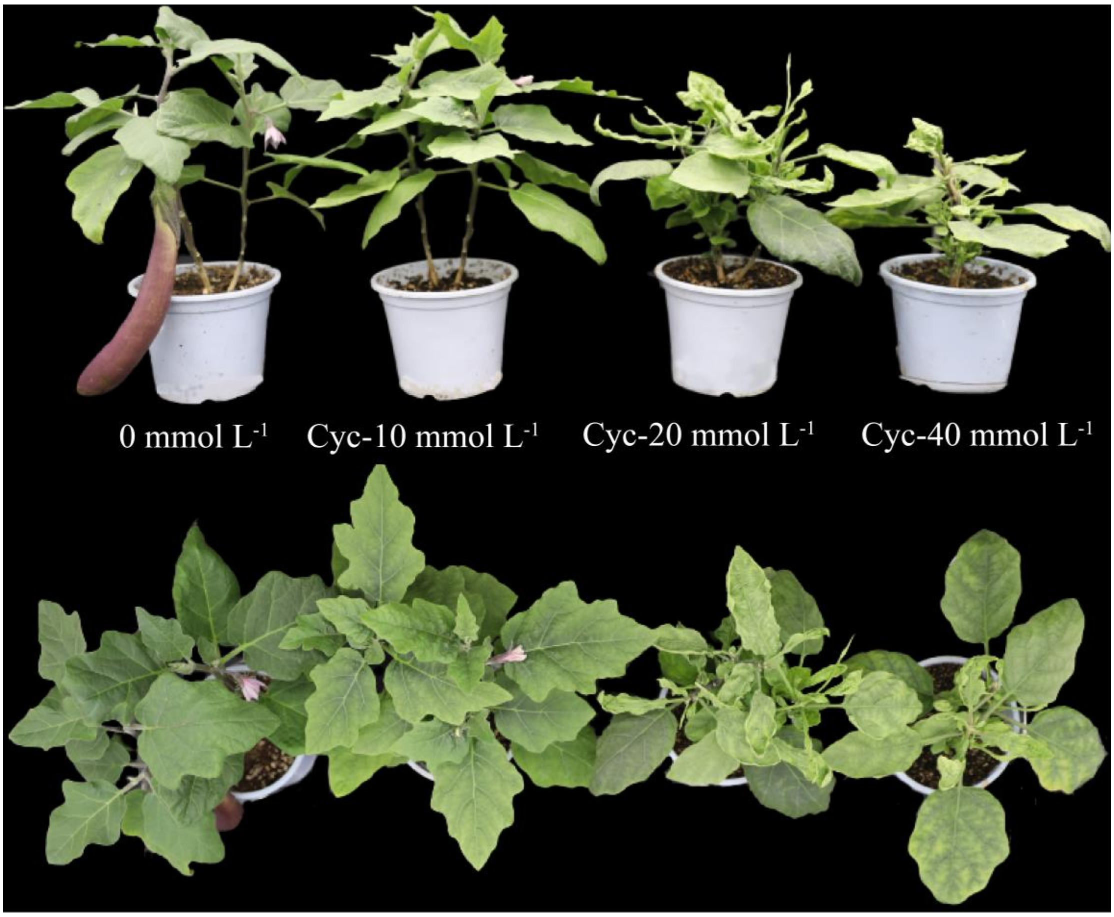

Figure S1. Effects of exogenous cyclic leucine spraying on growth and development of eggplant (day 30 after the end of spraying)
